# RETRACTION: Association Between Malnutrition and Surgical Site Wound Infection Among Spinal Surgery Patients: A Meta‐Analysis

**DOI:** 10.1111/iwj.70293

**Published:** 2025-03-03

## Abstract

**RETRACTION:** XieJ. , DuY. , TanZ. , and TangH. , “Association Between Malnutrition and Surgical Site Wound Infection Among Spinal Surgery Patients: A Meta‐Analysis,” International Wound Journal 20, no. 10 (2023): 4061–4068, 10.1111/iwj.14297.37391942 PMC10681542

The above article, published online on 30 June 2023, in Wiley Online Library (http://onlinelibrary.wiley.com/), has been retracted by agreement between the journal Editor in Chief, Professor Keith Harding; and John Wiley & Sons Ltd. Following an investigation by the publisher, all parties have concluded that this article was accepted solely on the basis of a compromised peer review process. The editors have therefore decided to retract the article. The authors did not respond to our notice regarding the retraction.

**RETRACTION:** J. Xie , Y. Du , Z. Tan , and H. Tang , “Association Between Malnutrition and Surgical Site Wound Infection Among Spinal Surgery Patients: A Meta‐Analysis,” International Wound Journal 20, no. 10 (2023): 4061–4068, 10.1111/iwj.14297.37391942 PMC10681542

The above article, published online on 30 June 2023, in Wiley Online Library (http://onlinelibrary.wiley.com/), has been retracted by agreement between the journal Editor in Chief, Professor Keith Harding; and John Wiley & Sons Ltd. Following an investigation by the publisher, all parties have concluded that this article was accepted solely on the basis of a compromised peer review process. The editors have therefore decided to retract the article. The authors did not respond to our notice regarding the retraction.

